# The Association between Sugar-Sweetened Beverages and Male Pattern Hair Loss in Young Men

**DOI:** 10.3390/nu15010214

**Published:** 2023-01-01

**Authors:** Xiaojin Shi, Hsiaohan Tuan, Xiaona Na, Haibing Yang, Yucheng Yang, Yulin Zhang, Menglu Xi, Yuefeng Tan, Celi Yang, Junhan Zhang, Ai Zhao

**Affiliations:** 1 Vanke School of Public Health, Tsinghua University, Beijing 100084, China; 2 Department of Dermatology, School of Clinical Medicine, Beijing Tsinghua Changgung Hospital, Tsinghua University, Beijing 102218, China

**Keywords:** sugar-sweetened beverages, male pattern hair loss, adult, male, youth

## Abstract

We performed this study to investigate the association between sugar-sweetened beverage (SSB) consumption and male pattern hair loss (MPHL) in young men. We conducted this cross-sectional study from January to April 2022 in mainland China. Young people aged 18–45 years (*n* = 1951) were recruited from 31 provinces in China. We used a self-reported online survey for data collection. We explored the associations between the amount/frequency of SSB consumption and MPHL by using a binary logistic regression model, with adjustments for sociodemographic, hair status, dietary intake, lifestyle, and psychological factors. Among the 1028 participants (27.8 ± 7.2 years) in the final analysis, we found that high SSB consumption is associated with a higher risk of MPHL. We recommend more support to decrease SSB consumption among young people to minimize negative health outcomes.

## 1. Introduction

Male pattern hair loss (MPHL), a progressive and non-scarring form of hair loss, has become a global public health problem. The incidence of MPHL continues to increase while the age of onset for MPHL continues to decrease. Based on previous surveys of the Chinese population, the MPHL prevalence has increased from 21.3% in 2010 to 27.5% in 2021 [1,2]. MPHL distresses young people by affecting an individual’s sense of self, causing psychological distress and adversely affecting quality of life [3,4,5,6]. MPHL is related to numerous factors, including genetics, anxiety, sleep time, age, body mass index (BMI), disease history, physical activities, nutrition, and smoking [7,8,9,10,11,12,13,14]. Dietary intake is considered to play an important role, and numerous studies have indicated the negative effect of the Western diet on MPHL [15,16]. As a major feature of the Western diet, the high consumption of added sugars might influence hair loss by triggering polyol pathways [17,18,19,20].

Sugar-sweetened beverages (SSBs), the consumption of which is prevalent in young populations [21,22,23,24], are any liquids that are sweetened with various forms of added sugars. SSBs include sodas/soft drinks, juice with added sugar, sport drinks, energy drinks, sweet milk, and sweet tea/coffee [25]. In the USA, 63% of youths and 49% of adults drink an SSB on a given day [24]. Research in China has also reported that SSB consumption is highest in the 13–29-year-old age group (22.38%) [23]. Current studies indicate that excessive SSB consumption is associated with chronic diseases, obesity, tooth decay, and emotional problems [26,27,28,29,30,31,32,33]. As a major dietary source of added sugars [34], SSBs may be a potential risk factor for MPHL.

However, epidemiological studies on the association between SSB intake and MPHL are still insufficient, especially among the young population. With limited data, a cross-sectional study indicated that sugary beverage intake is a protective factor for MPHL [9], which is inconsistent with the results of biological mechanism studies. Therefore, the association between SSB intake and MPHL needs to be confirmed by additional studies.

We aimed to explore the association between the frequency and amount of SSB intake and the MPHL status through an epidemiological study, to provide scientific evidence to improve the dietary habits and promote the health of young people.

## 2. Materials and Methods

### 2.1. Study Design and Participants

**Figure 1:**
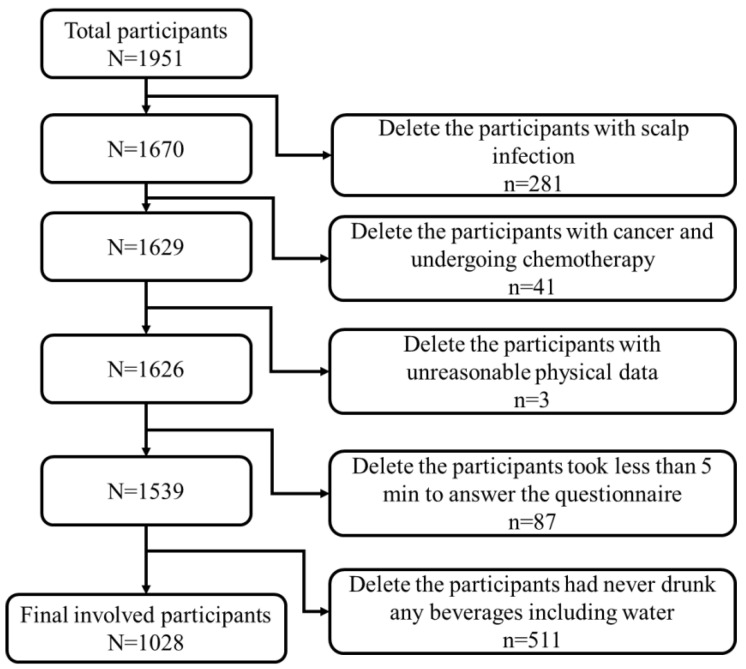
Flowchart of data selection.

We performed a cross-sectional study and collected data from January to April 2022. The participants were invited via a sampling strategy that combined multistage sampling and snowball sampling, through the Wenjuanxing e-questionnaire platform (Wenjuanxing Tech Co., Ltd., Changsha, China). We used the invitation letter to find potential study college students and teachers aged 18–45 years old living in South, North, and Central China. We subsequent reached additional participants by using a “snowball sampling” method [35]. In the invitation letter, we described the study purposes and content. At the beginning of the e-questionnaire, the participants had to click the option of “I agree to participate this study” before they could answer the questions. All data were collected anonymously. A total of 1951 Chinese males responded to the survey. Our study participants were recruited from 31 provinces, covering most geographic regions in mainland China. The inclusion criteria were (1) male, (2) aged 18–45 years old, and (3) currently living in mainland China. The participants who met the inclusion criteria received a reward after completing the survey. To ensure the survey quality, we added two attention-check questions to the survey. If the participants selected the wrong options for either question, it indicated poor data quality and we excluded their data from the study. To avoid repetition, IP restriction was applied, which meant that the survey could only be completed once from a single IP address. Furthermore, participants (1) with a scalp infection, (2) with cancer and undergoing chemotherapy, (3) with unreasonable physical data (height ≥ 2.5 m or ≤1 m, weight ≥ 300 kg or ≤10 kg), (4) took less than 5 min to answer the questionnaire, and (5) had never drunk any beverages including water were excluded, resulting in a total of 1028 usable responses for subsequent data analysis (Figure 1).

### 2.2. Data Collection

In the e-questionnaire, all the questions were grouped into five sections: (1) basic socio-demographic information, (2) hair status, (3) dietary intake, (4) lifestyles, and (5) psychological status. Each participant self-reported his weight and height. We calculated BMI by dividing body weight (kg) by the square of body height in meters (m^2^). According to the classification of weight status for Chinese adults [36], obesity, overweight, normal weight, and underweight are classified as BMI ≥ 28, 24–27.9, 18.5–23.9, and ≤18.5 kg/m^2^, respectively.

We used the Basic and Special (BASP) scale, established by Lee et al. [18], to evaluate the MPHL status. The BASP scale classifies the types of patterns of hair loss (PHL) for men. The basic (BA) types represent the shape of the anterior hairline, and the specific types (SP) represent the density of hair on distinct areas (frontal and vertex). There are four basic types (L, M, C, and U) and two specific types (F and V). The final type is decided by combining the assigned basic and specific types. One of the basic types must be selected, and the specific type may be selected if it exists. Each of the various types is subdivided into 3 or 4 grades, according to its severity. According to the guideline for diagnosis and treatments of androgenetic alopecia [37], we divided the participants into normal (L, M0, C0) and MPHL (M1~3, C1~3, V1~3, F1~3, U1~3) groups based on the final BASP types. The participants were asked “Have you been diagnosed with hair loss and received clinical treatment?”. We classified the participants based on whether they had received treatment. The participants were also asked about their hair care habits, including whether they had dyed, permed, bleached, or relaxed their hair in the prior week, and their hair washing frequency.

SSB consumption was determined from responses to the updated version of 15-item Beverage Intake Questionnaire (BEVQ-15), which we used to assess the participants’ usual consumption habit of beverage in the past 1 month [38]. We evaluated intake frequencies and amounts of 15 beverages, of which sweetened juice beverages, soft drinks, energy and sports drinks, sweetened milk, sweetened nut milk, sweetened tea beverages, and sweetened tea and coffee are considered SSBs. Based on the BEVQ-15, we divided SSB consumption into seven categories: never or <1 time/week, 1 time/week, 2–3 times/week, 4–6 times/week, 1 time/day, 2 times/day, and >3 times/day. Using the classification method of a previous study [39], we selected three nodes (1, 4, and 7 times/week) as the cut-off points to divide SSB intake into four groups with average weekly intake frequency being: [0, 1), [1, 4), [4, 7), and [7, 103) times/week. SSB intake at each time was recorded in milliliters (mL) for each reported beverage. We calculated the amount of SSB intake based on the frequencies and the amounts at each time. For analysis, we converted the amount categories for each beverage item into an equivalent weekly intake as follows: large amount (>3500 mL per week), moderate amount (1500~3500 mL/week), small amount (1~1500 mL/week), and never (0 mL/week).

The Food Frequency Questionnaire (FFQ) evaluates the consumption of 12 food groups: cereals; roots and tubers; vegetables; fruits; meat, poultry and offal; eggs; fish and seafood; pulses, legumes, and nuts; oils and fats; deep-fried food; sugar and honey; sweets and ice cream. The food groups originated from the Chinese Dietary Guideline and the Chinese Food Pagoda [40].

To assess physical activity, we used the International Physical Activity Questionnaire-Short Form (IPAQ-S). According to the World Health Organization (WHO) recommendations, we divided physical activity status into three levels: insufficient, adequate, and excessive. We considered adequate physical activity as: [1.25, 2.5] hours/week vigorous-intensity physical activity or [2.5, 5] hours/week moderate-intensity physical activity, or any equivalent combination of activity in a week [41]. Participants with insufficient or excessive physical activity performed less or more physical activity per week, respectively.

For smoking status, we divided participants into non-smokers, former smokers, or current smokers. We defined a never smoker as someone who had never smoked.

We used the Pittsburgh Sleep Quality Index (PSQI) to measure sleep duration, which we calculated as (wake time − sleep time) − (sleep latency + wake after sleep onset).

We also collected the chronic disease history and family history of baldness. Chronic disease history included diabetes, hyperglycemia, hypertension, hyperlipidemia, heart disease, thyroid diseases, kidney disease, liver disease, anemia, infections, asthma, food allergy, psoriasis, and seborrheic dermatitis. We asked participants about alopecia in first-, second-, and third-degree relatives.

We also evaluated psychological characteristics, including anxiety and post-traumatic stress disorder (PTSD). We used the Chinese version of the General Anxiety Disorder-7 (GAD-7) to evaluate anxiety status. This instrument assesses the occurrence of seven core symptoms over the past 2 weeks. The response options use a Likert scale ranging from 1 (never) to 3 (nearly every day). According to validation and standardization of the GAD-7 [42,43,44], we divided the anxiety status into minimal (0–4), mild (5–9), moderate (10–14), and severe (15–21). We estimated the PTSD status by using the Chinese version of the PTSD Checklist-Civilian Version (PCL-C), which consists of 17 items. Response options range from 1 (not at all) to 5 (seriously), and the total score ranges from 17 to 85. In this study, we used a score of ≥41 as the cut-off to identify PTSD symptoms [45].

### 2.3. Ethics

The questionnaire was completed anonymously. Informed consent was required prior to completing the survey by clicking the “agree” option to confirm willingness to participate voluntarily in the survey. The online survey was conducted in full agreement with the national and international regulations in compliance with the Declaration of Helsinki (2000). This study was approved by the institution review board of Tsinghua University in 2021 (project number: 20210178).

### 2.4. Statistical Analysis

We used R version 4.1.3 for data analysis. We compared means and proportions of different characteristics by using t-test for continuous variables and the chi-square test for categorical variables. Prior to hypothesis testing, we investigated the influence of confounding variables. Therefore, we generated a directed acyclic graph (DAG) with the online tool DAGitty version 3.0 (http://www.dagitty.net/dags.html (accessed on 10 October 2022)) [46]. The graph is presented in Figure S1; it provides a visual representation of the conceptual framework underpinning our analyses. In the multivariate analysis, binary logistic regression was applied and we adjusted the variables with statistical significance in univariate analysis and those variables that, although not statistically significant, play an important biological role. Model 1 had no adjustment for confounders. In model 2, we adjusted for age, education level, BMI, behavioral variables, disease history, hair care habits, and family history. In model 3, we also adjusted for all “food intake” variables significant in univariate analysis. The final model 4 is based on model 3, with adjustment for PTSD.

We generated linear (outcome are continuous variables) and logistic regression models (outcome are categorical variables) to assess the potential mediation effect of disease history and anxiety on the association of SSB intake frequency and the MPHL status. According to the procedure of Baron and Kenny [47], three conditions have to be met in a series of regression models to establish mediation: (1) the predictor is significantly associated with the outcome, (2) the predictor is significantly associated with the mediator, and (3) the mediator is significantly associated with the outcome when controlling for the predictor. If a mediation relationship exists, then the effect of SSB intake frequency on the MPHL status would be expected to decrease significantly after accounting for psychological characteristics. We examined indirect effects by using a non-parametric, bias-corrected bootstrapping procedure. The bootstrapping procedure generates an empirical approximation of the sampling distribution of the product of the estimated coefficients in the indirect paths using 5000 resamples from the data set.

To determine the robustness of the associations found using logistic regression analyses, we conducted sensitivity analyses (1) by excluding participants who had received clinical treatment for MPHL, and (2) by excluding participants with PTSD. All comparisons were bilateral, and we considered *p* < 0.05 to be statistically significant.

## 3. Results

The final dataset included 1028 male individuals comprising 436 (42.4%) normal participants and 592 (57.6%) participants with MPHL. Table 1 illustrates the sociodemographic and MPHL-related distribution of the participants. The mean age of the participants was 27.8 years (standard deviation [SD] = 7.2). Compared with normal individuals, those with MPHL were more likely to: be older, current or former smokers, and drinkers; have a lower education level, less physical inactivity, and a shorter sleep duration; have experienced severe anxiety and PTSD; have received clinical treatment for hair loss; have MPHL-related diseases, a positive family history of MPHL, and dyed/permed/bleached/relaxed hair.

The differences in dietary intake frequencies in the past month among participants with and without MPHL are presented in Table 2. These differences are significant based on the univariate analysis, except for consumption of cereals, roots and tubers, fruits, and fish and seafood. Compared with the normal group, the MPHL group consumed more deep-fried food, sugar and honey, and sweets and ice cream, and consumed fewer vegetables.

In total, nearly half of the participants (*n* = 459, 44.6%) stated that they consumed SSBs more than once a day (Table 3), 25.1% consumed SSBs 4–7 times a week, and 18.5% consumed SSBs 1–3 times a week. More than 10% of participants had not consumed SSBs over the prior month. The average weekly SSB intake was 4293 mL in the MPHL group, much higher than that in the normal group (2513 mL) (t = −3.645, *p* < 0.001). There was a significant difference for each of the four levels of SSB intake frequencies (amounts) between participants with and without MPHL. Compared with the normal group, the MPHL group consumed more SSBs.

The odds ratios (ORs) of SSB intake frequency with MPHL are listed in Table 4. Based on the crude model (model 1), compared with participants who never drank SSBs, those who drank more than 7 times/week are more likely to have MPHL, with an OR of 3.36 (95% confidence interval (CI) = 2.22, 5.09). Based on models 2 and 3, adjusted for age, education level, smoking status, alcohol intake, BMI, disease history, family history, hair dyeing/perming/bleaching/relaxing, sleep time, physical activity, and nutrient intake, the ORs and 95% CIs decrease to some extent but remain significant. However, in the final model (model 4), additionally adjusted for PTSD, the *p* value is not significant, with an OR of 1.57 (95% CI = 0.94, 2.64). The ORs of SSB intake amount with MPHL are not substantially different.

**Figure 2:**
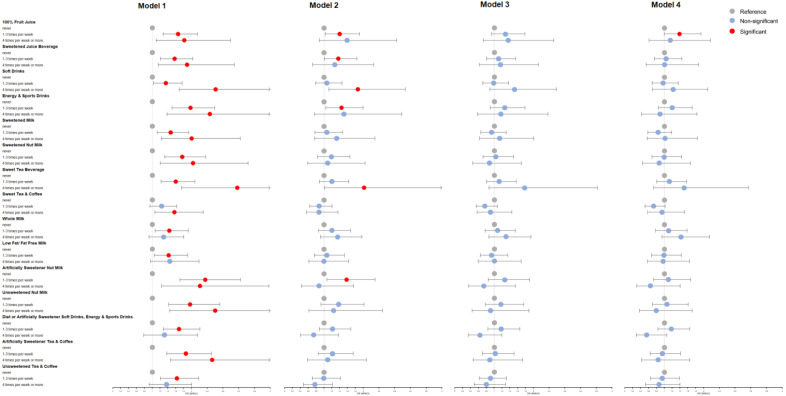
Association between different types of beverage intake frequency and male pattern hair loss (MPHL). Model 1 had no adjustment for confounders; Model 2 adjusted: age, education level, BMI, smoking status, alcohol intake, family history, hair dyeing/perming/bleaching/relaxing, sleep time and physical activity; Model 3, based on model 2, further adjusted consumption of vegetables, meats, eggs, beans and bean products, oils and fats, deep fried food, sugar and honey, sweets and ice cream; Model 4, based on model 3, further adjusted PTSD.

**Figure 3:**
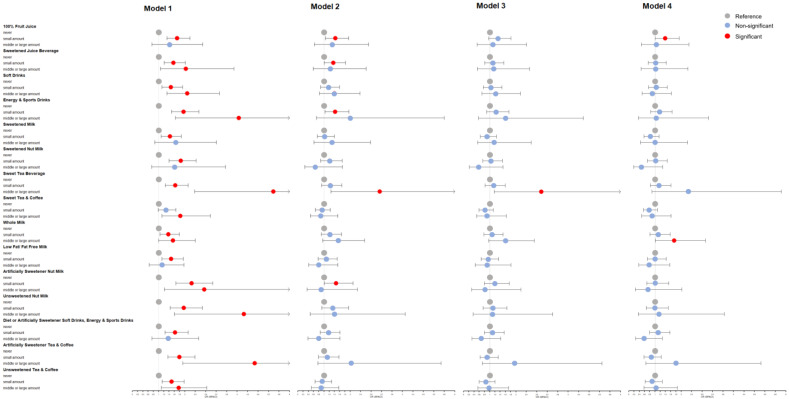
Association between different types of beverage intake amount and male pattern hair loss (MPHL). Model 1 had no adjustment for confounders; Model 2 adjusted: age, education level, BMI, smoking status, alcohol intake, family history, hair dyeing/perming/bleaching/relaxing, sleep time and physical activity; Model 3, based on model 2, further adjusted consumption of vegetables, meats, eggs, beans and bean products, oils and fats, deep fried food, sugar and honey, sweets and ice cream; Model 4, based on model 3, further adjusted PTSD.

We used forest plots to visualize the binary logistic regression results of different types of beverages (Figure 2 and Figure 3). In model 1, every type of SSB is significantly associated with MPHL as a risk factor. After adjusting for confounders, most SSBs still have a tendency to increase the risk of MPHL, especially juice beverages, soft drinks, energy and sports drinks, and sweetened tea beverages, which remain significant in model 2. Regarding beverages without added sugar, artificially sweetened nut milk and pure fruit juice are also potential risk factors for MPHL.

**Figure 4:**
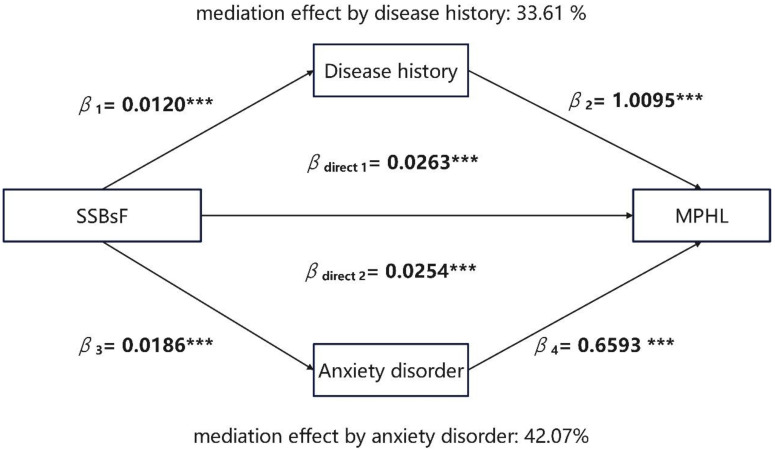
Mediation effect diagram for disease history and anxiety disorder in association between Sugar-Sweetened Beverages (SSBs) intake frequency and male pattern hair loss (MPHL). Anxiety disorder status was divided into four levels according to GAD-7. Score range included: 0–4 minimal anxiety, 5–9 mild anxiety, 10–14 moderate anxiety, 15–21 severe anxiety. Disease history was divided into three groups: without disease, one chronic disease and more than one disease. SSBsF means SSB intake frequency, which was included as a continuous variable in the mediation analysis. MPHL was divided into two groups according to BASP scale: normal group and MPHL group. *** *p* < 0.001.

In addition, we conducted mediation analysis using anxiety disorder status and disease history as mediators (Figure 4). SSB intake frequency significantly predicts MPHL, and the mediator disease history is significantly associated with SSB intake frequency and significantly affects MPHL. The contribution of disease history to MPHL is significant. The bootstrapping analyses showed that the indirect effect is significant (β_indirect_ = 0.0121, 95% CI = [0.0077, 0.0180]). Thus, disease history mediates the association between SSB intake frequency and MPHL. Similarly, there are significant associations between SSB intake frequency and anxiety disorder, and between anxiety disorder and MPHL. Moreover, the indirect effect of anxiety disorder is significant (β_indirect_ = 0.0122, 95% CI = [0.0083, 0.0170]). We can infer that anxiety disorder status mediates the association between SSB intake frequency and MPHL.

In the sensitivity analysis, we repeated the logistic regressions described above after excluding the data of participants with a clinical treatment history (Table S1) or a history of PTSD (Table S2). We observed similar results after excluding participants who had received clinical treatment for MPHL. However, the association in models 2 and 3 loses statistical significance when participants with PTSD are excluded.

## 4. Discussion

We aimed to understand the association between SSB consumption and the MPHL status among young men in China. We observed a high consumption level of SSB in the studied population and, for the first time, have reported that high consumption of SSB is associated with a higher risk of MPHL. Moreover, we found similar associations for different types of SSBs.

SSB consumption among the young population has increased dramatically in recent decades. According to data from 2003 to 2014, the annual per capita consumption of beverages in China increased from 12 to 119 kg [48], and the consumption continued to grow from 2014 to 2016 [49]. In a recent survey, the proportion of deaths caused by excessive consumption of carbon-containing beverages increased 35% from 1990 to 2019 [50]. In our study, only 11.8% of participants reported they had not consumed SSBs in the last month. The average intake frequency was 11.15 times per week, and the average intake amount was 3538.71 mL per week. One of the reasons for the high SSB intake in the young population is unawareness of the harmful effects of SSBs, although there is a large number of previous studies reporting the adverse effects of sugary beverages on health, such as mortality, cardiovascular disease, obesity, and dental caries [51,52,53]. A qualitative study indicated that many other factors influence the perceptions of beverage healthfulness, such as the color and transparency of the beverage packaging [54]. Chronic diseases and deaths are so vague and distant for young people that they are unwilling to give up the satisfaction brought by SSBs for the sake of long-term health goals. Therefore, further exploration of the possible and tangible health consequences of SSB in youth could help reduce SSB intake.

Interestingly, we found a significant association between high SSB consumption and MPHL. Several potential direct and indirect mechanisms could explain this association. Referring to the direct effect of SSBs on MPHL, the high sugar content in SSBs leads to a higher serum glucose concentration, which triggers the polyol pathway by creating a high affinity for aldose reductase [55]. The biochemical symptoms of androgenetic alopecia (AGA) in the scalp are highly suggestive of an overactive polyol pathway [55]. With a continuous glucose supply, the polyol pathway is reinforced by a positive feedback loop [56]. In vitro and in vivo studies have shown that glucose utilization in the polyol pathway reduces the amount of glucose available to the outer root sheath keratinocytes of hair follicles, and gluconeogenesis is also antagonized by depletion of ATP and phosphate levels [19,57]. Lack of energy in outer root sheath keratinocytes is considered a possible cause of MPHL. In addition, excessive sugar intake is often accompanied by excessive lipid intake, and a high-fat diet is also considered to be related to MPHL. Animal studies have shown that a high-fat diet can induce hair loss in mice [58]. However, after we adjusted the intake frequency of oils, fat, and deep-fried food, the association between SSBs and MPHL is still significant, indicating that SSBs are an independent factor associated with MPHL.

In addition to confounders, other MPHL-related factors may also play a mediating role. It is possible that the effect of excessive SSB intake on MPHL is mediated by chronic diseases and emotional problems. One of the most frequently mentioned type of hair loss in chronic diseases in relation to SSBs is AGA, especially in patients with diabetes, hypertension, hyperglycemia, thyroid dysfunction, and anemia [59,60,61,62,63,64]. Hair loss is generally regarded as the clinical manifestation of these diseases. Moreover, SSB intake is thought to promote emotional problems [30,31]. A cohort study confirmed an adverse effect of sugar intake from sweetened food and beverage on long-term psychological health [65]. According to a meta-analysis, participants who drank the equivalent of three cans of cola per day had an approximately 25% higher risk of depression than SSB nondrinkers [66]. A cross-sectional study found that the higher mean consumption of added simple sugars is significantly associated with higher anxiety among 45-year-old participants [67]. Those emotional problems might induce MPHL. A systematic review and meta-analysis found a significant association between emotions and AGA [68]. Based on our mediation analysis, we also confirmed that chronic diseases and emotional factors (anxiety status) act as mediator for the association between SSB intake and MPHL.

In addition to anxiety, other psychological factors such as PTSD may affect hair loss. A nationwide population-based cohort study indicated that the patients with PTSD had an increased risk of developing autoimmune skin diseases (ASDs), including alopecia areata (AA) [69]. Our univariate analysis showed that PTSD is significantly associated with MPHL. After adjusting for PTSD as a confounder in model 4, the association between SSB intake and MPHL is no longer significant. To provide more robust data, we conducted a sensitivity analysis excluding PTSD cases, and we still noted the association between SSB and MPHL. These findings imply that PTSD might be an important factor affecting MPHL, and the impact is stronger than SSB intake. It should also be noted that PTSD may be linked to SSB consumption. A pilot study showed that the added sugar consumption of older veterans with PTSD exceeds the U.S. dietary guideline recommendations [70]. The associations between SSB, PTSD, and hair loss require further confirmation.

To the best of our knowledge, there has been only one study conducted to explore the association between SSB intake and hair loss, and the results are inconsistent with our study. The previous web-based investigation indicated that men who consume sugary drinks are less likely to experience MPHL [9]. We speculate that the inconsistent results come from the method used to classify SSBs. The previous study did not distinguish the types of SSBs or specify the types of sweeteners added to the beverages and did not fully consider potential confounders. Indeed, different types of SSBs have distinct sugar contents and additives, so the effects might be different. Therefore, we examined the association between MPHL and intake of various types of SSBs.

We distinguished between different subtypes of beverages as well as the sources of sweetness: sugar and artificial sweeteners. Figure 2 and Figure 3 show that almost all the SSB subtypes have an adverse effect on MPHL, and this trend remains after adjusting for confounders, although the sample size of a single type is too small to produce statistical significance. As the only SSB subtype with a protective trend after adjustment, sweet tea and coffee are rich in caffeine, which plays a protective role in hair loss and alleviate the negative effect of added sugar [71]. In addition, artificially sweetened beverages have a significant association with MPHL, which alerts people to the negative effects of non-nutritive sweeteners on hair growth.

We have focused on the association between SSB consumption and MPHL in the young population, a topic that has rarely been explored. We have considered as many factors as possible, tested the robustness of the findings, and preliminarily explored the mechanism. However, there are several limitations to this study that should be addressed. First and foremost, it was a cross-sectional study, relying on self-reported data; thus, it is difficult to establish the temporal and causal relationships between SSB intake and MPHL, and recall bias may exist. Whether SSB overconsumption affects MPHL or MPHL affects participants’ SSB consumption requires further clarification. The use of an online survey might have excluded certain populations such as those with limited access to the internet and a lower education level. Moreover, the multistage sampling method was not randomized, which might have introduced selection bias. In addition, we did not distinguish the severity of MPHL because few of the participants had moderate and severe MPHL; thus, we cannot determine the effect of SSB intake on the severity of MPHL. Because MPHL evaluation was self-reported rather than clinically diagnosed, the results of this study may only have a certain suggestive effect. Finally, we did not collect data regarding the consumption of other sweetened products. Thus, we cannot address the exact effects of total sugar consumption on MPHL, but we have adjusted the frequency of sugar, honey, sweets, and ice cream consumption as confounders.

## 5. Conclusions

Reducing SSB consumption has become a thorny problem, puzzling governments and health institutions around the world. We have shown high SSB consumption in young Chinese people aged 18–45 years old, and those who consumed excessive SSB consumption had a higher likelihood of reporting MPHL. Anxiety disorder status and disease history might mediate the association between SSB consumption and MPHL. Emphasizing that SSB consumption could have a potential negative effect on one’s appearance could catch the attention of the young population and promote a reduction in SSB intake. Additional longitudinal and interventional studies are needed to confirm the current association and to provide information for evidence-based health education.

## Figures and Tables

**Table 1:** Basic characteristics of participants according to Basic and Special (BASP) types.

Characteristics		Overall (*n* = 1028)	BASP		*p*-Value
			Normal (*n* = 436)	MPHL (*n* = 592)	
**Socio-demographic characteristics**					
Age, year (M ± SD)		27.8 (±7.2)	26.5 (±6.5)	28.7 (±7.5)	<0.001
BMI, *n* (%)					0.094
	<18.5	88 (8.6)	40 (45.5)	48 (54.5)	
	18.5–23.9	678 (66.0)	301 (44.4)	377 (55.6)	
	24–27.9	170 (16.5)	58 (34.1)	112 (65.9)	
	28 or higher	92 (8.9)	37 (40.2)	55 (59.8)	
Education level, *n* (%)					0.006
	Senior high school and below	298 (29.0)	125 (41.9)	173 (58.1)	
	Undergraduate	616 (59.9)	247 (40.1)	369 (59.9)	
	Graduate and above	114 (11.1)	64 (56.1)	50 (43.9)	
Smoking status, *n* (%)					<0.001
	never smoker	485 (47.2)	278 (57.3)	207 (42.7)	
	former smoker	219 (21.3)	76 (34.7)	143 (65.3)	
	current smoker	324 (31.5)	82 (25.3)	242 (74.7)	
Alcohol intake in past month, *n* (%)					<0.001
	No	515 (50.1)	271 (52.6)	244 (47.4)	
	Yes	513 (49.9)	165 (32.2)	348 (67.8)	
Weekly physical activity time, *n* (%)					0.085
	0	197 (19.2)	68 (34.5)	129 (65.5)	
	0–2.5 h	124 (12.1)	53 (42.7)	71 (57.3)	
	2.5–5 h	263 (25.6)	114 (43.3)	149 (56.7)	
	more than 5 h	444 (43.2)	201 (45.3)	243 (54.7)	
Daily sleep time, *n* (%)					<0.001
	6 h or less	140 (13.6)	36 (25.7)	104 (74.3)	
	more than 6 h	888 (86.4)	400 (45)	488 (55)	
**MPHL related factors**					
GAD-7, *n* (%) ^a^					<0.001
	0–4	538 (52.3)	294 (54.6)	244 (45.4)	
	5–9	259 (25.2)	99 (38.2)	160 (61.8)	
	10–14	146 (14.2)	35 (24)	111 (76)	
	15–21	85 (8.3)	8 (9.4)	77 (90.6)	
PTSD, *n* (%) ^b^					<0.001
	No	648 (63.0)	353 (54.5)	295 (45.5)	
	Yes	380 (37.0)	83 (21.8)	297 (78.2)	
Received clinical treatment of hair loss, *n* (%)					<0.001
	No	991 (96.4)	435 (43.9)	556 (56.1)	
	Yes	37 (3.6)	1 (2.7)	36 (97.3)	
Disease history, *n* (%) ^c^					<0.001
	No	768 (74.7)	392 (51)	376 (49)	
	One chronic disease	124 (12.1)	28 (22.6)	96 (77.4)	
	More than one disease	136 (13.2)	16 (11.8)	120 (88.2)	
Family history, *n* (%) ^d^					<0.001
	No	812 (79.0)	368 (45.3)	444 (54.7)	
	Yes	216 (21.0)	68 (31.5)	148 (68.5)	
Hair dyeing/perming/bleaching/relaxing, *n* (%)					<0.001
	No	773 (75.2)	393 (50.8)	380 (49.2)	
	Yes	255 (24.8)	43 (16.9)	212 (83.1)	
Hair washing frequency, *n* (%)					0.339
	<3 times/week	181 (17.6)	71 (39.2)	110 (60.8)	
	≥3 times/week	847 (82.4)	365 (43.1)	482 (56.9)	

^a^ GAD-7 is the generalized anxiety disorder 7-Item scale. Score range included: 0–4 minimal anxiety, 5–9 mild anxiety, 10–14 moderate anxiety, 15–21 severe anxiety. ^b^ PTSD was estimated by PCL-C. PCL-C is the civilian version of PTSD check list. >41 means PTSD positive, ≤41 means PTSD negative. ^c^ Disease history included diabetes, hyperglycemia, hypertension, hyperlipidemia, heart disease, thyroid diseases, kidney disease, liver disease, anemia, infections, asthma, food allergy, psoriasis, and seborrheic dermatitis. ^d^ Family history included the participants have one or more blood relatives diagnosed with alopecia.

**Table 2:** Differences of dietary intake frequencies in past month among participants with and without male pattern hair loss (MPHL).

Characteristics		Overall (*n* = 1028)	BASP		*p*-Value
			Normal (*n* = 436)	MPHL (*n* = 592)	
Consumption of cereals, *n* (%)					0.079
	never	33 (3.2)	19 (57.6)	14 (42.4)	
	<3 times/week	472 (45.9)	190 (40.3)	282 (59.7)	
	4–6 times/week	207 (20.1)	81 (39.1)	126 (60.9)	
	≥7 times/week	316 (30.7)	146 (46.2)	170 (53.8)	
Consumption of roots and tubers, *n* (%)					0.322
	never	120 (11.7)	58 (48.3)	62 (51.7)	
	<3 times/week	637 (62.0)	271 (42.5)	366 (57.5)	
	4–6 times/week	176 (17.1)	66 (37.5)	110 (62.5)	
	≥7 times/week	95 (9.2)	41 (43.2)	54 (56.8)	
Consumption of vegetables, *n* (%)					0.001
	<3 times/week	457 (44.4)	167 (36.5)	290 (63.5)	
	4–6 times/week	241 (23.4)	101 (41.9)	140 (58.1)	
	≥7 times/week	330 (32.1)	168 (50.9)	162 (49.1)	
Consumption of fruits, *n* (%)					0.315
	never	41 (4.0)	17 (41.5)	24 (58.5)	
	<3 times/week	489 (47.6)	194 (39.7)	295 (60.3)	
	4–6 times/week	194 (18.9)	84 (43.3)	110 (56.7)	
	≥7 times/week	304 (29.6)	141 (46.4)	163 (53.6)	
Consumption of meat, poultry and offal, *n* (%)					<0.001
	never	88 (8.6)	51 (58)	37 (42)	
	<3 times/week	471 (45.8)	163 (34.6)	308 (65.4)	
	4–6 times/week	225 (21.9)	90 (40)	135 (60)	
	≥7 times/week	244 (23.7)	132 (54.1)	112 (45.9)	
Consumption of eggs, *n* (%)					0.003
	never	38 (3.7)	18 (47.4)	20 (52.6)	
	<3 times/week	523 (50.9)	193 (36.9)	330 (63.1)	
	4–6 times/week	211 (20.5)	105 (49.8)	106 (50.2)	
	≥7 times/week	256 (24.9)	120 (46.9)	136 (53.1)	
Consumption of fish and seafoods, *n* (%)					0.140
	never	192 (18.7)	88 (45.8)	104 (54.2)	
	<3 times/week	705 (68.6)	287 (40.7)	418 (59.3)	
	4–6 times/week	101 (9.8)	51 (50.5)	50 (49.5)	
	≥7 times/week	30 (2.9)	10 (33.3)	20 (66.7)	
Consumption of pulses, legumes, and nuts, *n* (%)					0.004
	never	125 (12.2)	71 (56.8)	54 (43.2)	
	<3 times/week	682 (66.3)	272 (39.9)	410 (60.1)	
	4–6 times/week	151 (14.7)	60 (39.7)	91 (60.3)	
	≥7 times/week	70 (6.8)	33 (47.1)	37 (52.9)	
Consumption of oils and fats, *n* (%)					0.002
	never	165 (16.1)	87 (52.7)	78 (47.3)	
	<3 times/week	503 (48.9)	189 (37.6)	314 (62.4)	
	4–6 times/week	139 (13.5)	55 (39.6)	84 (60.4)	
	≥7 times/week	221 (21.5)	105 (47.5)	116 (52.5)	
Consumption of deep-fried food, *n* (%)					<0.001
	never	249 (24.2)	134 (53.8)	115 (46.2)	
	<3 times/week	634 (61.7)	263 (41.5)	371 (58.5)	
	4–6 times/week	120 (11.7)	33 (27.5)	87 (72.5)	
	≥7 times/week	25 (2.4)	6 (24)	19 (76)	
Consumption of sugar and honey, *n* (%)					0.001
	never	332 (32.3)	170 (51.2)	162 (48.8)	
	<3 times/week	542 (52.7)	210 (38.7)	332 (61.3)	
	4–6 times/week	118 (11.5)	43 (36.4)	75 (63.6)	
	≥7 times/week	36 (3.5)	13 (36.1)	23 (63.9)	
Consumption of sweets and ice cream, *n* (%)					0.002
	never	264 (25.7)	137 (51.9)	127 (48.1)	
	<3 times/week	613 (59.6)	247 (40.3)	366 (59.7)	
	4–6 times/week	117 (11.4)	40 (34.2)	77 (65.8)	
	≥7 times/week	34 (3.3)	12 (35.3)	22 (64.7)	

**Table 3:** Sugar-sweetened beverages (SSBs) consumption in past month among participants with and without male pattern hair loss (MPHL).

Characteristics		Overall (*n* = 1028)	BASP		*p*-Value
			Normal	MPHL	
SSB intake frequency, *n* (%)					<0.001
	Never	121 (11.8)	73 (60.3)	48 (39.7)	
	1–3 times per week	190 (18.5)	103 (54.2)	87 (45.8)	
	4–7 times per week	258 (25.1)	117 (45.3)	141 (54.7)	
	more than 7 times per week	459 (44.6)	143 (31.2)	316 (68.8)	
SSB intake amount, *n* (%) ^a^					<0.001
	Never	121 (11.8)	73 (60.3)	48 (39.7)	
	Small amount	368 (35.8)	169 (45.9)	199 (54.1)	
	Middle amount	290 (28.2)	119 (41.0)	171 (59.0)	
	Large amount	249 (24.2)	75 (30.1)	174 (69.9)	

^a^ SSB intake amount included 4 levels: Never = 0 mL/week; Small amount = no more than 1500 mL/week; Middle amount =1500~3000 mL/week; Large amount = more than 3500 mL/week.

**Table 4:** Odds ratio and 95% confidence interval of MPHL among different SSB intakes.

		MPHL/All Cases	Model 1 ^a^	Model 2 ^b^	Model 3 ^c^	Model 4 ^d^
**SSB intake frequency**						
	Never	48/121	1.00 (Reference)	1.00 (Reference)	1.00 (Reference)	1.00 (Reference)
	1–3 times per week	87/190	1.28 [0.81,2.04]	1.13 [0.68,1.88]	1.15 [0.68,1.93]	1.21 [0.71,2.06]
	4–7 times per week	141/258	1.83 [1.18,2.84]	1.56 [0.96,2.54]	1.33 [0.80,2.23]	1.26 [0.74,2.12]
	>7 times per week	316/459	3.36 [2.22,5.09]	2.03 [1.26,3.27]	1.78 [1.07,2.95]	1.57 [0.94,2.64]
**SSB intake amount**						
	Never	48/121	1.00 (Reference)	1.00 (Reference)	1.00 (Reference)	1.00 (Reference)
	Small amount	199/368	1.79 [1.18,2.72]	1.44 [0.91,2.29]	1.32 [0.81,2.13]	1.34 [0.82,2.19]
	Middle amount	171/290	2.19 [1.42,3.37]	1.66 [1.02,2.71]	1.43 [0.85,2.39]	1.35 [0.80,2.27]
	Large amount	174/249	3.53 [2.24,5.55]	1.78 [1.06,2.98]	1.58 [0.92,2.74]	1.33 [0.76,2.32]

^a^ Model 1 had no adjustment for confounders; ^b^ Model 2 adjusted: age, education level, BMI, smoking status, alcohol intake, family history, hair dyeing/ perming/ bleaching/ relaxing, sleep time and physical activity; ^c^ Model 3, based on model 2, further adjusted consumption of vegetables, meats, eggs, beans and bean products, oils and fats, deep fried food, sugar and honey, sweets and ice cream; ^d^ Model 4, based on model 3, further adjusted PTSD.

## Data Availability

The datasets generated and analyzed during the current study are not publicly available because informed consent was not obtained for data sharing. However, the data are available from the authors upon reasonable request.

## Supplementary Materials

The following supporting information can be downloaded at: https://www.mdpi.com/article/10.3390/nu15010214/s1, Table S1: Association between SSB and MPHL in participants without clinical treatment; Table S2: Association between SSB and MPHL in participants without PTSD; Figure S1: Direct Acyclic Graph for the association of SSB intake and MPHL status in China, created using DAGitty version 3.0.
