# Supplementary material for: The Association between Sugar-Sweetened Beverages and Male Pattern Hair Loss in Young Men
Source: Nutrients. 2023 Jan 1;15(1):214. doi: 10.3390/nu15010214 (PMC9824121; doi:10.3390/nu15010214)
Supplement: Supplementary file 1 [file nutrients-15-00214-s001.zip › nutrients-2100692-supplementary.pdf]

Table S1. Association between SSB and MPHL in participants without clinical treatment.

	MPHL/all cases	Model 1	Model 2	Model 3	Model 4
**SSBs intake frequency**					
Never	45/118	1.00 (Reference)	1.00 (Reference)	1.00 (Reference)	1.00 (Reference)
1-3 times per week	81/184	1.28[0.80,2.05]	1.15[0.69,1.93]	1.15[0.68,1.97]	1.22[0.71,2.09]
4-7 times per week	137/253	1.92[1.23,2.99]	1.67[1.02,2.75]	1.43[0.85,2.41]	1.35[0.79,2.30]
>7 times per week	293/436	3.32[2.18,5.07]	2.09[1.29,3.40]	1.82[1.08,3.04]	1.60[0.95,2.71]
**SSBs intake amount**					
Never	45/118	1.00 (Reference)	1.00 (Reference)	1.00 (Reference)	1.00 (Reference)
Small amount	188/356	1.82[1.19,2.78]	1.51[0.94,2.42]	1.37[0.84,2.23]	1.39[0.84,2.30]
Middle amount	162/281	2.21[1.42,3.43]	1.75[1.07,2.86]	1.48[0.88,2.50]	1.40[0.82,2.39]
Large amount	161/236	3.48[2.19,5.53]	1.81[1.07,3.07]	1.61[0.92,2.81]	1.33[0.75,2.36]

Model 1 had no adjustment for confounders; Model 2 adjusted: age, education level, BMI, smoking status, alcohol intake, family history, hair dyeing/ perming/ bleaching/ relaxing, sleep time and physical activity; Model 3 based on model 2 further adjusted consumption of vegetables, meats, eggs, beans and bean products, oils and fats, deep fried food, sugar and honey, sweets and ice cream; Model 4 based on model 3 further adjusted PTSD.

Table S2. Association between SSB and MPHL in participants without PTSD.

	MPHL/all cases	Model 1	Model 2	Model 3
**SSBs intake frequency**				
Never	29/96	1.00 (Reference)	1.00 (Reference)	1.00 (Reference)
1-3 times per week	60/152	1.51[0.87,2.60]	1.29[0.72,2.34]	1.27[0.69,2.35]
4-7 times per week	85/172	2.26[1.33,3.83]	1.97[1.10,3.53]	1.59[0.86,2.96]
>7 times per week	121/228	2.61[1.57,4.34]	1.76[0.99,3.13]	1.48[0.80,2.76]
**SSBs intake amount**				
Never	29/96	1.00 (Reference)	1.00 (Reference)	1.00 (Reference)
Small amount	123/266	1.99[1.21,3.27]	1.69[0.97,2.91]	1.48[0.83,2.61]
Middle amount	84/177	2.09[1.23,3.53]	1.82[1.01,3.28]	1.47[0.78,2.74]
Large amount	59/109	2.73[1.53,4.85]	1.32[0.68,2.55]	1.23[0.61,2.48]

Model 1 had no adjustment for confounders; Model 2 adjusted: age, education level, BMI, smoking status, alcohol intake, family history, hair dyeing/ perming/ bleaching/ relaxing, sleep time and physical activity; Model 3 based on model 2 further adjusted consumption of vegetables, meats, eggs, beans and bean products, oils and fats, deep fried food, sugar and honey, sweets and ice cream.

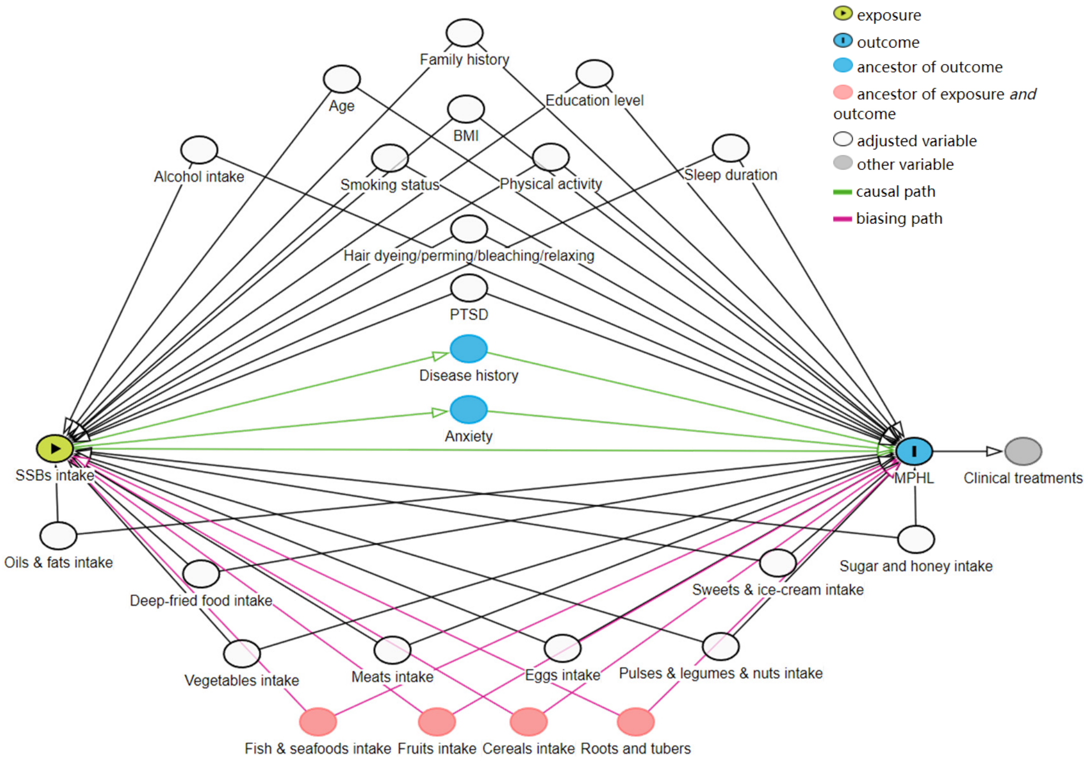

Figure S1. Direct Acyclic Graph for the association of SSBs intake and MPHL status in China, created using DAGitty version 3.0
